# Placenta accreta spectrum care has moved beyond subspecialty identity: A call to refocus the field on individualized, expertise‐driven systems of care

**DOI:** 10.1002/pmf2.70355

**Published:** 2026-07-09

**Authors:** Brett D. Einerson, Yalda Afshar, Daniela A. Carusi, John C. Markley, J. Newton, Christine Warrick, Lisa Zuckerwise, Jennifer Gilner, Sebastian R. Hobson, Karin A. Fox, Alireza A. Shamshirsaz, the Pan‐American Society for Placenta Accreta Spectrum (PAS2)

**Affiliations:** ^1^ Division of Maternal‐Fetal Medicine, Department of Obstetrics and Gynecology Utah Placenta Accreta Program University of Utah Health Salt Lake City Utah USA; ^2^ Advanced Maternal Hemorrhage and Accreta Care Program, Division of Maternal Fetal Medicine, Department of Obstetrics and Gynecology David Geffen School of Medicine University of California Los Angeles Los Angeles California USA; ^3^ Molecular Biology Institute University of California Los Angeles Los Angeles California USA; ^4^ Surgical Obstetrics & Abnormal Placentation Program, Department of Obstetrics & Gynecology Brigham & Women's Hospital, Harvard Medical School Boston Massachusetts USA; ^5^ Department of Anesthesia and Perioperative Care University of California San Francisco San Francisco California USA; ^6^ Division of Maternal‐Fetal Medicine, Department of Obstetrics and Gynecology Vanderbilt University Medical Center Nashville Tennessee USA; ^7^ Division of Obstetric Anesthesia, Department of Anesthesia Utah Placenta Accreta Program University of Utah Health Salt Lake City Utah USA; ^8^ Division of Maternal‐Fetal Medicine University of Virginia Charlottesville Virginia USA; ^9^ Complex Obstetric Surgery and Placenta Accreta Spectrum Care Program, Division of Maternal‐Fetal Medicine, Department of Obstetrics and Gynecology Duke University Durham North Carolina USA; ^10^ Division of Maternal Fetal Medicine, Department of Obstetrics & Gynaecology Mount Sinai Hospital, University of Toronto Toronto Ontario Canada; ^11^ Division of Maternal‐Fetal Medicine, Department of Obstetrics and Gynecology University of Texas Medical Branch Health Galveston Texas USA; ^12^ Fetal Care and Surgery Center, Division of Fetal Medicine and Surgery, Boston Children's Hospital Harvard Medical School Boston Massachusetts USA

*“Data are, thus, accumulating that reasonable hospital volume is a plausible predictor of outcome after surgery… Even if the skill of the individual surgeon is important, it seems to be even more critical that the multidisciplinary treatment team develops substantial experience in the management of the patients.”*

—Ingemar Ihse, MD, 2003, referring to cancer surgical care [1]

The recent report by Forrest and colleagues describing improved perioperative outcomes for placenta accreta spectrum (PAS) hysterectomies with maternal‐fetal medicine specialists as primary surgeons is the latest in a series of mostly single‐center analyses framed around the question of *which subspecialty should lead PAS surgery* [2]. Parallel work in gynecologic oncology literature has argued for the centrality of oncologic surgeons in PAS cases [3, 4]. While there is no doubt that, over the past decades, gynecologic oncologists have been the most technically appropriate surgeons to execute PAS cases in many obstetric centers, it is also clear that PAS care and cancer care are very different. The field's own evidence has been building toward a different approach for more than a decade: PAS outcomes are determined far more by program structure, institutional experience, and coordinated systems of care than by the subspecialty designation of the lead surgeon.

PAS is no longer an emerging condition—it is a well‐characterized, high‐risk diagnosis with a defined evidence base demonstrating that maternal outcomes improve when care is centralized, anticipatory, and multidisciplinary [5, 6, 7]. At the same time, we recognize that not every PAS case will be diagnosed antenatally. Even in mature programs with high‐quality imaging, some PAS cases remain unsuspected until delivery [8]. The need is therefore not only to optimize planned care but to build systems robust enough to manage both expected and unexpected cases. This viewpoint is offered as a call‐to‐action with three goals: to redirect the field from subspecialty‐based debate toward expertise‐based program accountability; to describe concrete strategies already in use to operationalize this shift; and to propose measurable actions that academic societies, programs, and individual clinicians can take to extend high‐quality PAS care to every patient who needs it.

## 1 THE EVIDENCE BASE: SYSTEMS OVER SUBSPECIALTY

Seminal work by Shamshirsaz and others demonstrated over a decade ago that a standardized multidisciplinary approach to PAS management—encompassing prenatal diagnosis, operative planning, anesthesia, blood bank coordination, and postoperative critical care—was associated with significant reductions in maternal morbidity compared with non‐standardized care [6, 7, 9]. These findings have been consistently replicated across institutions and care models [10, 11, 12], including in both expected and unexpected PAS cases [13, 14], firmly establishing multidisciplinary, protocolized care as the cornerstone of improved outcomes [5, 15, 16].

More recent data, including contemporary cohort analyses evaluating maternal‐fetal medicine specialists as primary surgeons within established PAS programs, further reinforce this principle [2]. Improvements in transfusion burden, intensive care utilization, and length of stay occur in the context of program maturation, earlier diagnosis, and fewer emergent procedures—not as a function of subspecialty alone. These findings should be interpreted not as evidence for or against any single discipline, but as confirmation that expertise embedded within a functional system is what saves lives.

## 2 THE PERSISTENCE OF THE SUBSPECIALTY QUESTION—AND WHAT IT TELLS US

Notwithstanding more than a decade of program‐level data, the question of which subspecialty should perform PAS surgery continues to surface in the literature and in institutional decision‐making. Some studies suggest that gynecologic oncology participation is associated with reduced complications [4]. Others, including contemporary program‐controlled analyses, have failed to demonstrate the benefit of any single subspecialty once program maturity and case characteristics are accounted for [3, 17]. These studies are limited by failing to define a PAS expert—one who has sufficient training and experience with PAS surgery. If such expertise is standardized, it is quite possible that controlling for this factor will override any perceived effect of classical subspecialty training. The arguments most frequently offered for the preference of one specialty—familiarity with complex open hysterectomy for gynecologic oncologists and some gynecologic surgeons, or expertise in high‐risk obstetric delivery and pregnancy anatomy for obstetricians and maternal‐fetal medicine specialists—reflect legitimate strengths. They do not, however, fully address the question since cancer surgery expertise and obstetric expertise alone do not confer the surgical fluency PAS demands.

## 3 WHAT ACTUALLY DRIVES EXCELLENCE: DEDICATION, DISCIPLINE, AND SYSTEMS

If fellowship training does not determine outcomes, what does? Three interconnected elements emerge as essential.

**Dedication** is the foundational commitment to mastering PAS care. It is the orientation that drives clinicians to seek additional training, engage with specialized literature and educational opportunities, consult colleagues at other experienced centers, and approach each case as an opportunity for learning. Dedication cannot be credentialed; it must be cultivated and sustained.

**Discipline** is the consistent application of best practices even when conditions are unfavorable. In PAS care, randomized data are sparse, and many practices are necessarily derived from a combination of evidence, accumulated experience, and multidisciplinary consensus. Discipline therefore means developing care pathways that integrate these inputs and applying them consistently. Hospitals offering PAS care need to build teams that understand and implement best practices when patients’ needs arise. Discipline also requires honest engagement with adverse outcomes—meticulous tracking, structured review, and systematic implementation of lessons learned.

**Systems** provide the organizational infrastructure within which dedication and discipline produce reliable results [18]. Effective PAS systems include reliable prenatal screening and experienced imaging interpretation; standardized surgical protocols developed through multidisciplinary consensus; readily available blood products with clear massive transfusion protocols; experienced anesthesia and nursing teams familiar with PAS management; operating room configurations optimized for complex pelvic surgery; written quality policies; structured self‐assessment; internal and external peer review; and participation in centralized data registries with feedback to participating centers. Systems are not synonymous with resources alone—they include the policies and review processes that turn resources into reliably good outcomes.

These three elements are mutually reinforcing. Dedication motivates individuals to acquire and refine expertise. Discipline ensures that this expertise is expressed consistently in clinical practice. Systems supply the organizational infrastructure that allows individual expertise to translate into reliable program‐level outcomes. None is sufficient on its own. High‐performing PAS programs achieve the integration of all three.

## 4 STRATEGIES DEMONSTRATING PROMISE

Important work is already underway across the international PAS community that operationalizes these principles. Multinational collaborative efforts coordinated by the Pan‐American Society for Placenta Accreta Spectrum (PAS^2^), the International Society for Placenta Accreta Spectrum (IS‐PAS), and International Federation of Gynecology and Obstetrics (FIGO) have begun to align research, training, and clinical practice across institutions and borders. Ongoing prospective studies are evaluating standardized approaches to diagnosis, preoperative planning, and treatment [19]. Formal training pathways such as the Toronto Complex Obstetric Surgery Fellowship offer structured PAS surgical education that transcends subspecialty boundaries. Simulation‐based training [20] and telemedicine networks [21] are connecting smaller programs to mature centers, expanding access to expertise without requiring patient transfer. Adaption of international guidelines for low‐ and middle‐income settings further illustrates that systems‐based PAS care is not confined to well‐resourced contexts [22].

## 5 IMPLEMENTATION REALITIES AND ADAPTIVE STRATEGIES

A systems‐based, expertise‐driven framework must be honest about the conditions in which it will be implemented. Many institutions cannot become high‐volume PAS referral centers. PAS is uncommon enough that genuine surgical volume is concentrated in relatively few programs, and persistent constraints—staffing, budget, declining trainee surgical volumes, and competing demands on clinical time—make universal access in every setting unrealistic. There are also places where multiple subspecialties cannot be assembled.

We advocate for a tiered, networked model. Every institution that performs deliveries should have a defined PAS response plan that includes either the ability to mobilize a surgical team with the required expertise or standing relationships with a regional referral program for PAS. Readiness for PAS outside a specialty referral center requires clear protocols for both emergent and non‐emergent referrals, intraoperative consultation, and management when transfer is not feasible. This model maximizes collaboration between high‐volume centers and referring hospitals and can ensure that PAS is not encountered in an unprepared system.

## 6 THE QUESTIONS THAT NOW MATTER

The substantive work of the field lies in answering key actionable questions.

***How do we define, measure, and credential PAS surgical expertise?*** Current approaches rely on fellowship certification and board eligibility, which serve as proxies for general competency but do not reflect actual PAS experience or outcomes. Existing formal training pathways demonstrate that cross‐specialty PAS training is feasible. The field needs standardized criteria for PAS surgical expertise that incorporate case volume, complexity of cases managed, patient outcomes, multidisciplinary collaboration, and continuous professional development.

***How do we individualize operative strategy based on placental adherence pattern, vascular anatomy, prior surgical history, and patient‐specific risk and preference?*** PAS encompasses a heterogeneous spectrum of pathology requiring tailored surgical approaches. Research must move beyond broad comparisons of surgical techniques or subspecialty designations toward precision‐medicine approaches that match operative strategy to individual patient characteristics and values.

***How do we quantify program readiness and institutional resources as modifiable determinants of outcome?*** Unlike individual surgeon characteristics, program‐level factors can be systematically improved through targeted interventions. Research identifying which program elements most strongly predict outcomes would enable evidence‐based quality improvement and inform accountability standards.

***How do we ensure equitable access to mature PAS programs as incidence continues to rise?*** Cesarean delivery rates remain elevated globally; PAS incidence will continue increasing. Not all patients can access high‐volume referral centers. Hub‐and‐spoke regional networks, telemedicine consultation, and society‐coordinated simulation training can extend reliable PAS care beyond the small number of centers that currently provide it.

## 7 A PATH FORWARD: CONCRETE ACTIONS FOR PROGRAMS AND THE FIELD

Translating these principles into accountability requires specific, measurable actions. We propose six:

1. Standardize PAS‐specific outcome measures, including PAS‐tailored definitions of severe maternal morbidity [23], to enable meaningful comparisons in research and quality improvement.
2. Adopt universal clinical definitions for PAS as the basis for case‐mix adjustment and classification, so that programs caring for higher‐acuity patients are compared on equitable terms.
3. Establish centralized, anonymized data registries with structured feedback to participating centers, enabling programs to identify and address performance gaps without punitive exposure.
4. Institutionalize structured case review, both within programs and through external peer assessment, so that adverse outcomes consistently drive iterative team learning.
5. Charge academic societies—PAS^2^, IS‐PAS, and FIGO—with developing accessible, PAS training pathways open to OBGYN clinicians from multiple backgrounds.
6. Build hub‐and‐spoke regional networks paired with telemedicine consultation to extend expertise to settings that cannot independently develop high‐volume PAS programs.

The evidence supporting this transition has been clear for more than a decade. Multidisciplinary programs improve PAS outcomes. Subspecialty designation of the primary surgeon does not independently predict outcomes once program factors are controlled. Volume, experience, systematic approaches, and coordinated care are what matter.

It is time for PAS care to fully reflect these realities. Collectively, we should now direct our effort toward building and sustaining programs that integrate expertise, dedication, discipline, and systems—so that the highest quality care reaches every patient with this challenging condition.

## AUTHOR CONTRIBUTIONS

**Lisa Zuckerwise**: Conceptualization; writing—review and editing. **Christine Warrick**: Conceptualization; writing—review and editing. **Brett D. Einerson**: Conceptualization; writing—review and editing; writing—original draft. **Alireza A. Shamshirsaz**: Conceptualization; writing—review and editing. **Yalda Afshar**: Conceptualization; writing—review and editing; writing—original draft. **Sebastian R. Hobson**: Conceptualization; writing—review and editing. **Daniela A. Carusi**: Conceptualization; writing—review and editing. **John C. Markley**: Conceptualization; writing—review and editing. **Jennifer Gilner**: Conceptualization; writing—review and editing. **Karin A. Fox**: Conceptualization; writing—review and editing.

## FUNDING INFORMATION

The authors received no specific funding for this work.

## CONFLICT OF INTEREST STATEMENT

The authors declare no conflicts of interest.

## Data Availability

The data that support the findings of this study are available from the corresponding author upon reasonable request.
